# Assessment of Subcortical Source Localization Using Deep Brain Activity Imaging Model with Minimum Norm Operators: A MEG Study

**DOI:** 10.1371/journal.pone.0059856

**Published:** 2013-03-20

**Authors:** Yohan Attal, Denis Schwartz

**Affiliations:** 1 CRICM UMR-S975 - Centre de Recherche de l’Institut du Cerveau et de la Moelle Epinière, Université Pierre et Marie Curie-Paris 6, Paris, France; 2 U975 Inserm, Paris, France; 3 UMR7225 CNRS, Paris, France; 4 ICM – Institut du Cerveau et de la Moëlle épinière, Paris, France; 5 CENIR – Centre de Neuro-Imagerie de Recherche, Paris, France; University College of London - Institute of Neurology, United Kingdom

## Abstract

Subcortical structures are involved in many healthy and pathological brain processes. It is crucial for many studies to use magnetoencephalography (MEG) to assess the ability to detect subcortical generators. This study aims to assess the source localization accuracy and to compare the characteristics of three inverse operators in the specific case of subcortical generators. MEG has a low sensitivity to subcortical sources mainly because of their distance from sensors and their complex cyto-architecture. However, we show that using a realistic anatomical and electrophysiological model of deep brain activity (DBA), the sources make measurable contributions to MEG sensors signals. Furthermore, we study the point-spread and cross-talk functions of the wMNE, sLORETA and dSPM inverse operators to characterize distortions in cortical and subcortical regions and to study how noise-normalization methods can improve or bias accuracy. We then run Monte Carlo simulations with neocortical and subcortical activations. In the case of single hippocampus patch activations, the results indicate that MEG can indeed localize the generators in the head and the body of the hippocampus with good accuracy. We then tackle the question of simultaneous cortical and subcortical activations. wMNE can detect hippocampal activations that are embedded in cortical activations that have less than double their amplitude, but it does not completely correct the bias to more superficial sources. dSPM and sLORETA can still detect hippocampal activity above this threshold, but such detection might include the creation of ghost deeper sources. Finally, using the DBA model, we showed that the detection of weak thalamic modulations of ongoing brain activity is possible.

## Introduction

Magnetoencephalography (MEG) and electroencephalography (EEG) are well known to have a high temporal resolution (a millisecond time scale) but a low spatial resolution for source localization compared to functional magnetic resonance imaging (fMRI) [1]. Moreover, this spatial resolution decreases rapidly as a function of the depth of the generators [2]. Thus, the detectability of deep brain structure activities is still an open question [3]–[7]. In addition to the larger sensors-to-source distance, subcortical brain sources in basal ganglia or in hippocampus have a complex cyto-architecture that could lead to the conclusion that their contribution to sensors is quasi-null. Nevertheless, quantifying the deeper neural currents at a higher time scale than fMRI is crucial for studying their implications in many brain processes (language, action, motor [8], [9] or emotion [10], [11]) and related disorders (stroke, epilepsy, Alzheimer’s, Parkinson’s diseases [12]). For the last two decades, more and more MEG and EEG (M/EEG) studies have reported activations that were generated by neural generators in the hippocampus, amygdala or basal ganglia (see [13] for a review). Moreover, several papers based on realistic simulations [6], [14], [15] have studied the ability of beamformer or minimum current estimate solutions to localize hippocampal generators. These studies estimated the necessary signal strength, the minimum distance between the sources or numbers of trials, to localize the subcortical generators. They also evaluated the impact of the experimental paradigm and how data subtraction prior to source localization could improve the detection of weak hippocampal sources (below 5 nAm) [14].

Here, based on the results of [16], we make the hypothesis that subcortical contributions are not null, at least for the hippocampus, amygdala and thalamus. We focus on the assessment of the localization error of subcortical neural generators using the deep brain activity (DBA) model. DBA models the anatomical and electrophysiological properties of subcortical structures. These models are based on an imaging approach, which realistically distributes the current dipoles (CDs) over the neocortex and subcortical structures. CDs are usually assumed to model the synchronous summation of postsynaptic potentials that originate from neocortical macro-columns of pyramidal neurons. The DBA model introduces more electrophysiological knowledge by specifying a CD location, orientation and current density in a structure-specific manner.

To estimate the current distributions, we use three methods that are based on the widely used minimum L2 norm estimate (MNE) [17]. Because classical MNE gives source localization that is biased toward more superficial sources, activities of deeper generators are under-estimated. To alleviate this problem, it is possible to apply depth weighting [18], [19] to the MNE solution, obtaining a weighted minimum norm estimate (wMNE). Two other methods using noise-normalized depth-weighted MNE solutions are compared in this study, dynamic statistical parametric mapping (dSPM) [20] and standardized low-resolution electromagnetic tomography (sLORETA) [21]. Recently, Hauk et al. [22] assessed the differences between these inverse operators at the neocortical level. They show that depending on the goodness criteria and the experimenter’s specific questions, there is not a superior method within these three operators. In this study, we want to compare the characteristics of the three operators in the specific case of subcortical generators. These linear approaches naturally produce a smooth source localization estimate that makes it difficult to assess the localization accuracy with typical error distances. The question of choosing good error metrics is not trivial, and several studies proposed different metrics [19], [22], [23]. Based on these studies, our ability to localize deep currents using DBA is assessed by two dipole localization error (DLE_s_) metrics:

DLE_g_ corresponds to the Euclidian distance of a solution’s gravity center from the true location.
DLE_m_ corresponds to the Euclidian distance of a solution’s maximum from the true location.

To quantify the source localization accuracy of our framework, the paper is designed as follows. First, the forward model, which is built using the anatomies of seven subjects, is assessed in terms of the sensitivity (the sum of squares for the gain vector of each source) and the intensity of the simulated fields. This part tells us how the neural architecture of subcortical structures influences the amplitude of the resulting magnetic fields by studying their distributions compared to the cortex contribution. We then quantify the point-spread functions (PSF) and the cross-talk functions (CTF) using the resolution matrix [19], [24]–[26] in the specific case of the hippocampus. PSF quantifies the distortion of point source reconstruction by the inverse operators. CTF quantifies the distortion that is induced from other source locations. Using these two measures, we assess the regional distortion impact of one subcortical source on other cortical and subcortical sources and the influence of these other sources on a given subcortical source. Second, we perform Monte Carlo simulations [19], [27], [28]. Using the MEG resting state activity from the seven subjects, which is considered to be additional noise, we simulate MEG signals from subcortical activations across all of the structures of our model, using an increasing size for the activated patches. In a second step, we add a neocortical activation of 3 cm^2^ in the visual area. Temporal delays between cortical and subcortical activations range from instantaneous to entirely shifted activations. It allows us to modulate the overlap ratio between the cortical and the subcortical neural currents when we quantify the minimum subcortical amplitude that is required for detection, compared to a cortical amplitude. To summarize, we address two main questions. First, we assess our ability to detect a single subcortical activation under optimal conditions. Second, we quantify the minimum ratio of subcortical activation to neocortical activation that allows good subcortical detection. Finally, as a practical illustration, we use our DBA model to analyze a resting state experiment. This last step is intended to answer the following question: Can we detect, by using alpha power modulation, the activity in the thalami that has no specific neural organization (in contrast to the neocortical macrocolumn of pyramidal cells) and a very deep location?

## Methods

### 2.1 DBA Model Setup

DBA defines a model of neural generators that is based on anatomical and electrophysiological priors for neocortex and subcortical structures. To solve the forward and inverse problem and to generate simulated fields, the framework is based on the following key points:

An accurate anatomical model to constrain the global source space.
An electrophysiological model to constrain dipole orientations.
A realistic current dipole moment density (DMD) to activate patches of CDs over these structures.

We describe, hereafter, the key points of the setup; the complete framework is detailed in [13], [16].

The anatomical model that corresponds to the source space location is computed on individual T1-weighted MRI volume data (3T Siemens Magnetom VERIO, 1 mm isotropic resolution, axial scans) from 7 healthy subjects. The neocortical sheet composes the global anatomical model together with the amygdalo-hippocampal complex and several central grey nuclei and related structures (putamen, thalamus, reticular perithalamic nucleus (RPN), lateral geniculate nucleus (LGN) and external pallidum (EGP)). The model pipeline creation is based on the works of Chupin et al. [29] and Yelnik et al. [30] and uses the Brainvisa software [31] http://brainvisa.info/.

**Figure 1:**
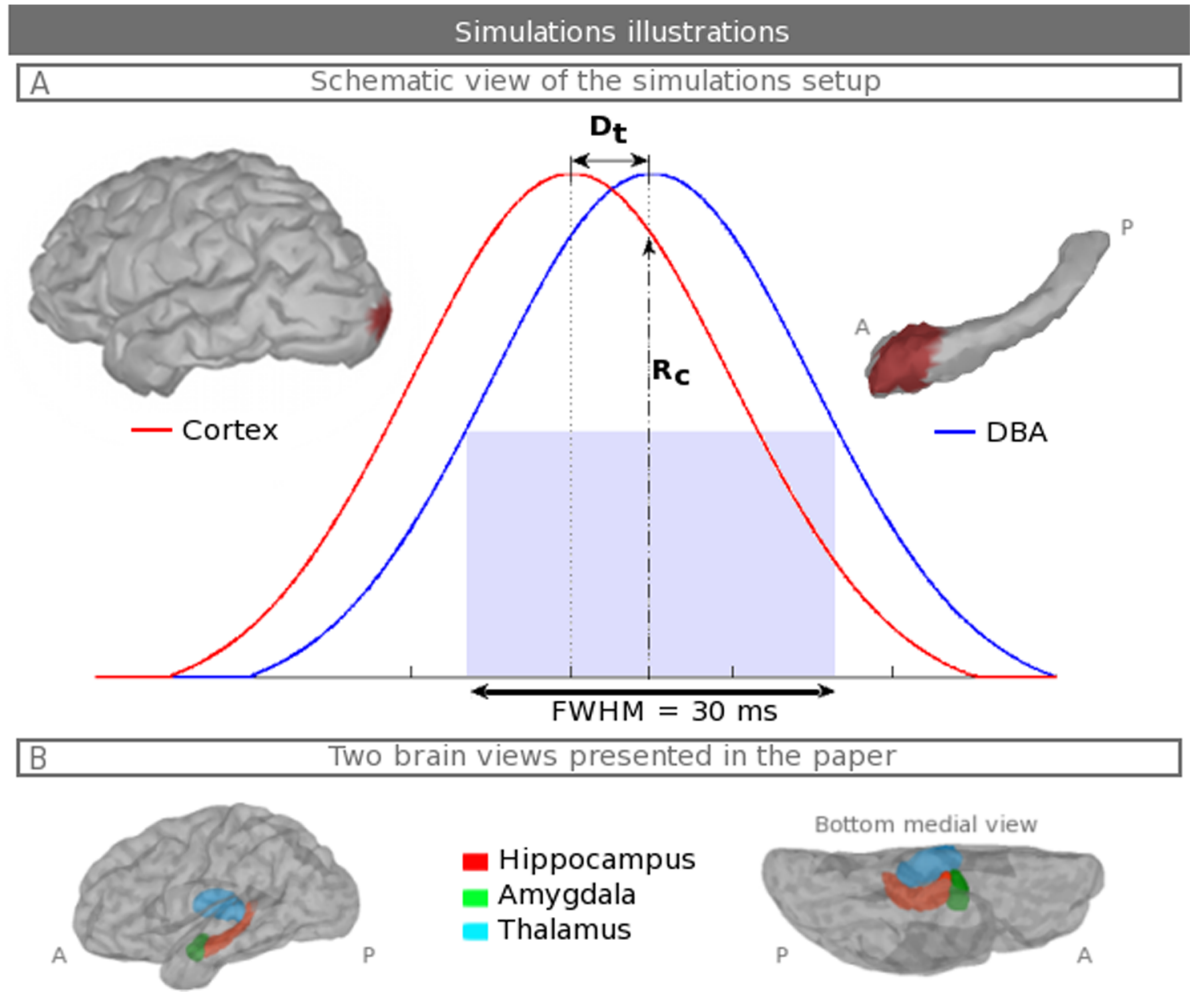
Simulation setup illustrations. A. Schematic view of the simulation setup. The red and blue Gaussians correspond to the distributions that modulate the neocortical (respectively, subcortical) simulated fields that are generated from the activation of patches; these fields are illustrated with neocortical and hippocampal tessellations. The summation of both activations is used to estimate the actual generators. D_t_ is the time between the maximum of the two Gaussians. The variation in D_t_ allows variations in the ratio R_c_ between the cortical and the subcortical activations. B. The lower part displays the anatomical model that has the mentioned structures and is included to give to the reader a better idea of their positions. “P” stands for posterior and “A” anterior.

The electrophysiological properties in the model are CDs distributed at each location defined by the global anatomical model. As usual, neocortical source orientations are constrained to the local normal of the cortical mantle at each vertex location of the gray-white matter interface. Central regions of the neocortical tessellation that correspond to the corpus callosum and residual brainstem parts from MRI segmentation are manually removed from the global source space. In contrast to the neocortex, large-scale electrophysiology of basal ganglia and related structures is better modeled by distributing current dipoles inside regular volume grids that are fitted within their surface envelopes. Indeed, the resulting currents from synchronous activities generated by sub-territories are generated by volumetric gray matter nuclei. DBA considers two types of neural generators, “open” and “closed” field cells (see Table 1), according to the resulting electromagnetic field produced by their dendritic arborization [32], [33]. For nuclei with an oriented neural architecture (EGP, RPN and LGN), dipoles are orientated along the principal axis of their respective surface envelope. The thalamus and striatum are essentially made of closed-field cells (i.e., with no preferred source orientation); hence, a current dipole is placed at each node of the inner volume grid with a random orientation [34]–[36]. In the specific case of the amygdala, its basolateral nucleus is mainly composed of pyramidal cells (i.e., open-field cells) without preferential orientation [37], [38]. Therefore, the amygdala is modeled in the same way as the thalamus and striatum [11]. The hippocampus is a complex structure in terms of the neural cells and architectural diversity. However, because we do not have access to the precise inner structure using 3T anatomical MRI, we limit the hippocampal source space to the external envelope (see Figure 1). CDs are distributed, similarly to the neocortex, orthogonally to the local surface. A first approximation assumes that pyramidal neurons that compose the 3-layered archeo-cortex [39] can be represented by a hippocampal tessellation. The characteristics of the global anatomical and electrophysiological source model are detailed in Table 1.

**Table 1:** Global characteristics of the DBA model.

Structures (Left)	Cortex	Hippocampus	Amygdala	Thalamus	LGN	EGP	Putamen	RPN
Surface | Volume(cm^2^ | cm^3^)	750	15	1	8	0.2	1.5	9	2
Number of vertices	4619	900	273	1043	229	453	1029	529
Cell type	O	O	O	C	O	O	C	O
DMD( 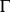 | 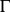 )	0.25	0.4	1	0.025	0.25	0.0025	0.25	0.0025
Neural current for patchsizes 1 to 5 ( 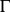 )	25 to 125	40 to 200	100 to 500	2.5 to 12.5	25 to 125	0.25 to 1.25	25 to 125	0.25 to 1.25

Anatomical and electrophysiological properties of the DBA model for left hemisphere structures (MNI template anatomy). Structures to the left of the vertical bar are considered to be surfaces, and structures to the right of the vertical bar are volumes. O stands for “open field” cells, and C stands for “closed field” cells. DMD stands for the dipole moment density, and the neural currents for the patch size are computed according to equation 1.

### 2.2 Subjects

Seven healthy volunteers (6 men and 1 woman, 30 years old on average) participated in this study. The local ethical committee approved the experimental procedures, and all of the participants gave written informed consent (CPP Ile-de-France VI, Groupe Hospitalier Pitié-Salpêtrière, n°7024). The MEG session is detailed in section 2.4.

### 2.3 Monte Carlo Simulations

Realistic simulated activations from subcortical structures are added to the individual data. Source simulations involve the sequential activation of surface patches or volume patches with increasing size at every source location (cortex, hippocampus and basal ganglia; see the illustration in Figure 1). For each structure, the number of simulations is set to the number of nodes in the corresponding source grid (see Table 1). Each patch of activation comprises a subset of connected vertices that belong to the source space. The simulations are performed for patch sizes ranging from 1 to 5 cm^2^ by steps of 1 cm^2^ for surface patches and 1 to 5 cm^3^ by steps of 1 cm^3^ for the volume patches; the simulations are limited to the left hemisphere of the brain. For the regions that have a total surface area that is lower than 5 cm^2^ and a volume lower than 5 cm^3^, such as the LGN, the higher patch size is defined as the total surface area (respectively, volume). The surface (respectively, volume) current strength 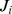 of the dipole *i* is computed as follows:
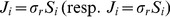
(1)


The surface area 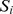 (or volume 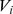) is associated with the dipole *i*, and 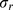 (respectively, 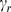) is the corresponding surfacic (respectively, volumetric) DMD in the region under consideration. DMD values, the resulting 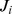 associated with the five sizes of patches and other characteristics of the DBA model are detailed in Table 1 (for more details about the electrophysiological assumptions, see [13]).

The gain matrix, *G*, that relates to the surface and volume source grids is computed using an overlapping spheres model implemented in the Brainstorm software [40] version 3.1, which is documented and freely available for download online under the GNU general public license (http://neuroimage.usc.edu/brainstorm). In the case of MEG, which is less sensitive than EEG to distortions from volume current diffusion, the lead field computation could be reasonably well approximated with an adapted spherical geometry compared to a realistic geometry [41]. Finally, the simulated field *M* is generated by the classical equation:
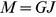
(2)


Simulated fields combining neocortical activation and subcortical activation result from the addition of both fields from equation (2). For each activated patch, the signal-to-noise ratio (the ratio between the energy of spontaneous activity at rest and the simulated activation energy) is equal to 20, which is an acceptable approximation of an evoked MEG response using ∼100 to 200 average trials. The neocortical activation is defined as a patch of 3 cm^2^ in the visual cortex and is used to simulate a visual stimulation in many experimental protocols. The subcortical activations are computed over all of the sources and for the five sizes of patches. A Gaussian distribution (FWHM = 30 ms) modulates both activations after a 200 ms baseline of eyes opened rest data. We introduce a variation of the temporal correlations between neocortical and subcortical activations. Neocortical and subcortical activations are separated in time by an interval D_t_ that ranges from 0 to 60 ms. D_t_ equaling 0 ms means that the activations appear at the same time. D_t_ equaling 60 ms means that the neocortical and subcortical activations are fully separated in time. Because we assess DLE_s_ at the maximum of the subcortical activation, D_t_ allows us to modulate the ratio, R_c_, of the neocortical activation added to the subcortical activation. Thus, R_c_ ranges from 100% to 0% (see the illustration in Figure 1).

### 2.4 Inverse Solutions and Localization Error Metrics

According to Maxwell’s equations, the measured magnetic fields are linear with respect to the dipole moment generated by neural generators and nonlinear with respect to the source locations [1]. Hence, it is convenient to separate the dipole moment into its magnitude and orientation parts to apply constraints on the locations and orientations from priors used in DBA. Finally, only the magnitude 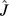 of current generators must be estimated by solving the inverse problem starting from equation (2), which is based here on the minimum L2 norm estimate:
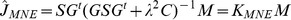
(3)where 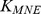 is the inverse operator, the superscript *t* indicates the matrix transpose, 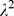 is the regularization parameter, and *C* and *S* are, respectively, the noise and the source covariance matrix. By depth weighting *S* such as 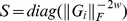, the depth bias of 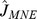 can be partially compensated [42]. Here, *w* is the weighting factor, and 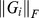 is the Frobenius norm of the gain matrix containing three dipole components for the *i-th* point source before applying orientations. Consequently, this depth weighting leads to the inverse operator wMNE on which we will focus this paper. Additionally, depth bias compensation and noise normalization are used by the two other inverse operators, sLORETA and dSPM. Both dSPM and sLORETA are derived from the inversion kernel 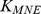. dSPM normalizes 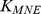 by the noise sensitivity (MNE of the noise) at each location [20], using the noise covariance matrix:
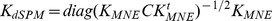
(4)sLORETA applies depth bias compensation and source standardization using the resolution matrix [21]. From equations (2) and (3), the resolution matrix R [19], [24], [25], [27] is obtained as:



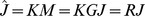
(5)Thus, the sLORETA inversion kernel is computed using 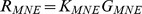:
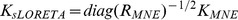
(6)


All inverse operators are computed with Brainstorm v3.1 (using a weighting factor of w = 0.6 and a regularization parameter of SNR = 3) and a baseline of 200 ms from the individual eyes opened rest activity to compute the noise covariance matrix.

PSF and CTF are obtained from the resolution matrix. Rows of *R* (CTF) quantify the point sources that induced modifications on other point sources. Columns of *R* (PSF) allow mapping the representation of a point source by a given inverse operator *K*, i.e., the induced distortions of the inverse operator. The lower the values of CTF and PSF are, the more accurate is the estimation. As shown in [22], we can see from equations (4) and (6) that only the rows of *R* are scaled by the normalization applied with the diagonal matrices, and the columns are not modified. Thus, the shape of CTF must not change across the operators, but the PSF will vary because of the normalization. For that reason, we will particularly focus on the PSF differences induced by the three kernels in terms of spatial dispersion and maximum mis-location. Based on the metrics used in [19], [22], [23], our ability to localize subcortical currents using DBA is assessed by two dipole localization error (DLE) metrics (in centimeters):

DLE_g_ corresponds to the Euclidian distance of a solution’s gravity center 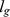 to the true location 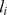:

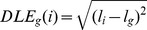
(7)

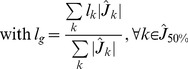
(8)

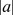
 represents the absolute value of a. 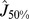 is the threshold estimated currents map containing dipoles having amplitudes that are above 50% of 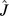
’s maximum, to remove weak sources.

DLE_m_ corresponds to the Euclidian distance of a solution’s maximum 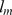 to the true location 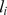:

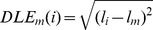
(9)DLE_m_ is widely used to quantify the mis-localization of the estimated maximum of the true source. This error assumes that the current map is distributed spherically around the maximum and that the chosen threshold is defined as a fraction of this maximum. However, this assumption is not always true, and DLE_m_ does not tell us how well the spatial extent of the estimated map is mapped. Using DLE_g_ as the gravity center of the estimation, Lin et al. propose to account for the classical Euclidian distance metric with the spatial extension. Given these two types of DLE_s_, the localization assessment becomes more understandable and accurate concerning the different properties of each method.

The group-level analysis across the seven subjects is performed using Brainstorm by the registration of each individual anatomy on the Montreal Neurological Institute (MNI) brain Template. Then, the individual maps (CTF, PSF, DLE) are projected on the template before a group average.

### 2.5 Thalamo-cortical Loop Using the Resting State MEG Experiment

We seek here to detect alpha power modulations in the thalamus during the well-established contrast between resting state eyes opened and eyes closed (EO/EC) [43], [44]. Alpha ongoing modulations are well known to be dominant during the eyes-closed condition, and neocortical alpha waves are widely considered to be paced by the thalamus [45], [46]. In fact, in the early seventies, it was clearly demonstrated that the cortical visual regions are significantly correlated with the thalamus during alpha rhythms [47]. Moreover, using multimodal EEG-fMRI, studies described thalamic modulations during the resting state [48]. The ability to detect thalamic activations using MEG was already shown by the early nineties with the work of [4], [49] and, recently, [50]. As mentioned at the beginning of the simulations setup, seven healthy volunteers participated in this study. The MEG session was composed of a block-designed EO/EC paradigm, with each block lasting for 30 s, for a total duration of 5 minutes per condition. MEG signals are recorded on a 151-channel CTF whole-head system, at a 1250 Hz sampling rate. The data are first band-pass filtered within the individual alpha band of each subject. For each subject, distributed source imaging is performed using the DBA model and the three inverse operators. Student t-tests are used to detect modulations of source amplitudes by contrasting the eyes opened vs. eyes closed conditions. All of the source maps are registered and normalized to the MNI reference template for group statistical analysis. Group average source maps are then thresholded at p<0.05 and FDR corrected [51], [52].

## Results

### 3.1 Sensitivity and Simulated Fields

**Figure 2:**
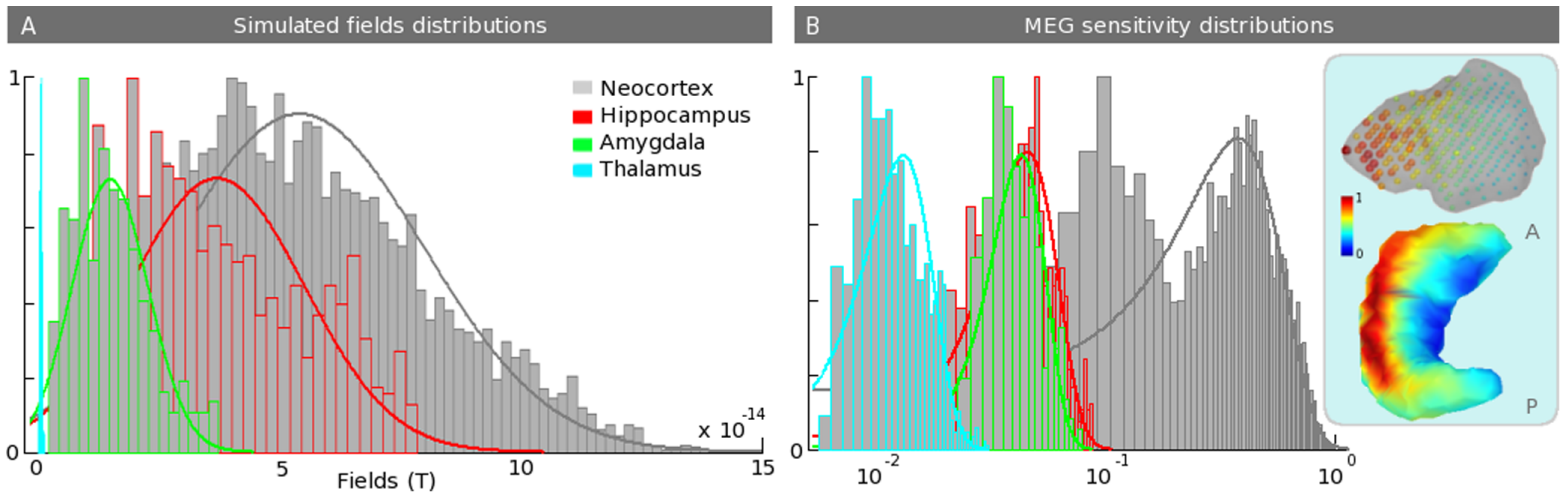
Simulated magnetic fields and sensitivity distributions. A. Plot of normalized distributions (with fitted Gaussian distributions) of the simulated fields for patches of 3 cm^2^ that belong to the four structures. These distributions account for the DMD of each structure, the geometry of the patches and the gain matrix of the sources that belong to the patches. B. Normalized averaged sensitivity distributions (normalized average root mean-squared (RMS) contribution to sensors) over 7 subjects. The corresponding maps are displayed for the hippocampus and the amygdala. Note that the x-axis is logarithmic and that the colormap is scaled at the subcortical level to better evaluate the distributions of the subcortical sources. These distributions are calculated using the gain vector at each source location.

Figure 2B shows the normalized grand averaged MEG sensitivity distributions (and fitted Gaussian distributions) that correspond to the normalized average RMS contribution to sensors for neocortex (gray), hippocampus (red), amygdala (green) and thalamus (light blue). The corresponding sensitivity maps for the hippocampus surface and the amygdala volume are also displayed. Figure 2A illustrates the normalized histogram (and fitted Gaussian distributions) for simulated fields, using patches of 3 cm^2^ that belong to the above-mentioned structures. As expected, the sensitivity to subcortical sources is more than ten times lower than that in the neocortex. However, considering an appropriate electrophysiological and anatomical model, the simulated fields are strong enough to overlap parts of the neocortical field’s distribution, except for the thalamus, which produces lower fields. We can see at the subcortical structures level (right blue rectangle) that there is a decreasing gradient of the sensitivity, starting from the parts of the structure that are closer to the sensors. However, the same pattern does not occur for the hippocampus tail. Note that the color map is scaled to the subcortical sensitivity. Furthermore, the sensitivity is lower on the hippocampus edges because they are mainly composed of radial sources. At the cortical scale (not shown in Figure 2), the sensitivity drops off rapidly with the distance to the sensors [2], with a lower sensitivity appearing on the gyri’s crests. Posterior sources have a stronger sensitivity because of the usual positions of the subjects within the MEG helmet (head resting on the back of the helmet). Note that the x-axis of the right graphic is logarithmic for a better evaluation of the distributions of the subcortical sources.

### 3.2 Point Spread and Cross-talk Functions

**Figure 3:**
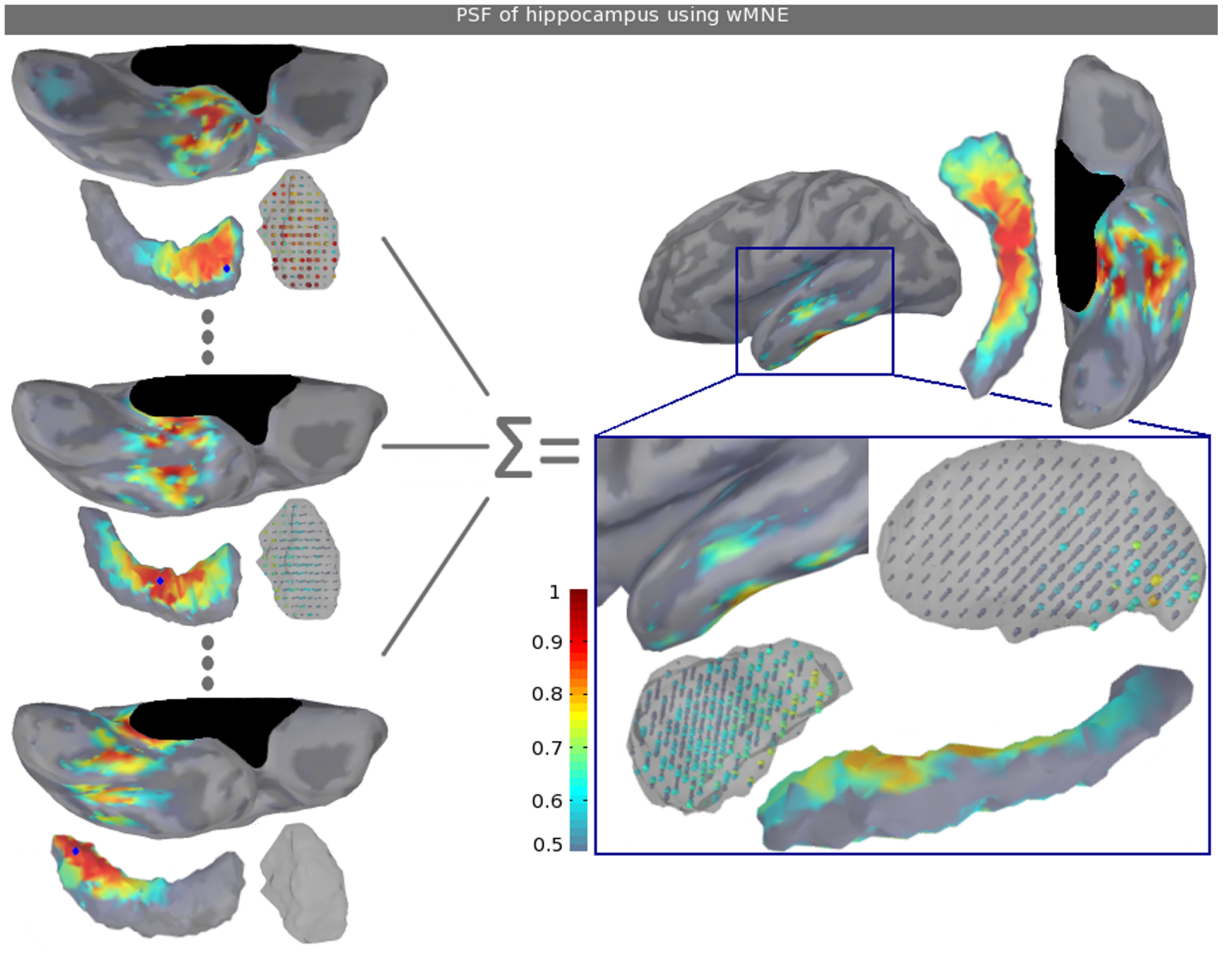
Point spread functions of hippocampus sources using wMNE. The left side displays the PSF maps of three point sources (blue dots) using wMNE. Each PSF map is normalized (i.e., normalization of the given point source’s column of the resolution matrix) to better visualize each spatial distribution. By averaging the PSF maps over all of the hippocampus and after normalization, we obtain the PSF maps that are displayed on the right side. Note that the colorbar does not start from zero and that the structure sizes are modified to make the sources more visible. See Figure 1B for a medial view of the relative position of the structures.

Figure 3 displays, on the left side, the PSF maps of three hippocampal sources (blue dots) in the case of the wMNE method. This PSF and CTF study is performed for the first subject and thresholded at 50% of the maximal value. The three sources are chosen in the head, the body and the tail. We can see the PSF moving according to this location. In the case of the head source, mainly the head but not the edges are affected. The distortion also extends into the nearest subcortical and neocortical regions. Strong PSF values are found in the amygdala, which is the closest region to this source, and in the anterior temporal lobe. In the two other cases, PSF values are lower in the amygdala and remain distributed in the nearest regions. On the right side, the resulting average PSF map of the overall hippocampal sources shows that the highest values are located in the medial and lateral temporal lobe, which are the closest regions of the hippocampus. More precisely, on the medial view, the highest values are found in the parahippocampal and entorhinal cortices. On the lateral view, the highest values are located in the temporal pole, especially on the crests of the superior and inferior temporal sulci. Among the seven subcortical structures that compose the model, the distribution extends into the thalamus and the amygdala, specifically into the regions that are closest to the hippocampus (see the zoom in the blue rectangle). Finally, hippocampal edges that are mainly composed of radial sources have no point spread.

**Figure 4:**
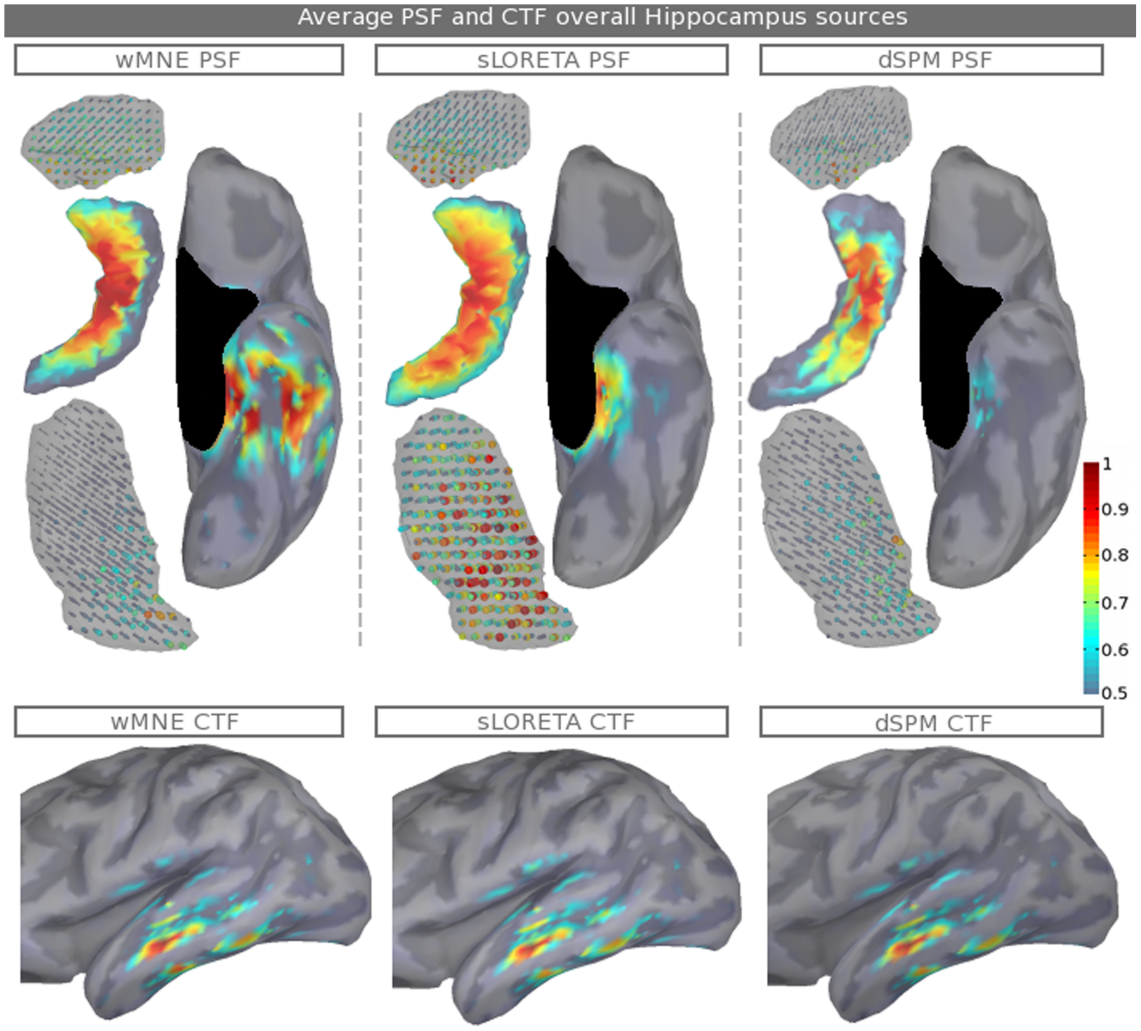
Average PSF and CTF maps over all hippocampus sources. Average PSF (1^st^ line) and CTF (2^nd^ line) maps are shown for the three inverse kernels: wMNE, sLORETA and dSPM. Note that the colorbar does not start from zero and that the structure sizes are modified to make the sources more visible. See Figure 1B for a medial view of the relative position of the structures.

Figure 4 aims to compare the average PSF and the average CTF maps (as presented in Figure 3) of the three inverse operators. Averaging is performed over all of the hippocampus sources; thus, the wMNE PSF map is identical to the map of Figure 3. The PSF and CTF maps are shown, respectively, on the 1^st^ and 2^nd^ lines. wMNE, sLORETA and dSPM are shown, respectively, on the 1^st^, 2^nd^ and 3^rd^ columns. The sLORETA PSF map shows a significant decrease in PSF in the neocortex but still shows significant values in the parahippocampal area. However, a PSF increase in the deeper regions is shown in the thalamus and the nearest amygdala part. dSPM PSF are small in the neocortex and decrease in the hippocampus and other subcortical structures. From the CTF point of view, the maps of the three methods are identical. This result is in agreement with the theory. Indeed, noise normalization is performed on the columns of the resolution matrix and, thus, does not modify the CTF [22]. The strongest values of the CTF maps are located in the lateral temporal lobe, especially in the superior temporal sulcus. There are no significant regional differences between source locations in the hippocampus concerning the CTF distribution.

### 3.3 Monte Carlo Simulations

**Figure 5:**
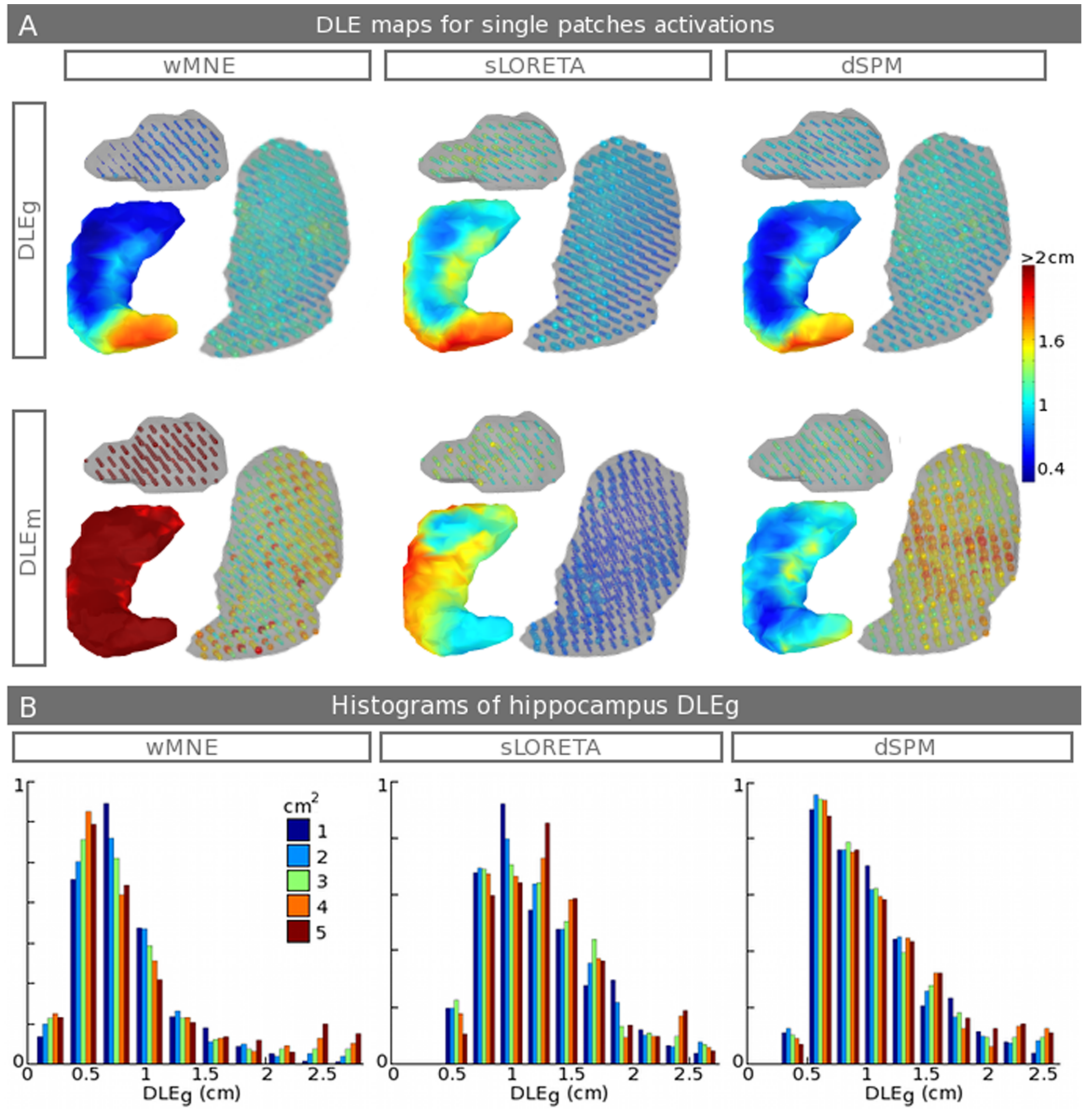
Monte Carlo Simulations of single activations. A. DLE_g_ (1^st^ line) maps and DLE_m_ (2^nd^ line) for single activations over the hippocampus, amygdala and thalamic sources and across the seven subjects. The results are shown for the three inverse methods, wMNE (1^st^ column), sLORETA (2^nd^ column) and dSPM (3^rd^ column). Note that the colorbar does not start from zero and that the structures sizes are modified to make the sources more visible. See Figure 1B for a medial view of the relative position of the structures. B. Histograms of DLE_g_ (x-axis in cm) distributions for all sizes of hippocampus patches (1 to 5 cm^2^). Note that the y-axes are normalized to be comparable.

Figure 5A shows the DLE_s_ maps averaged across the seven subjects for D_t_ = 60 ms, which correspond to single patch activations of 3 cm^2^ (or 3 cm^3^) for each surface (or volume) structure, respectively. Only the hippocampus, the amygdala and the thalamus results are presented here. DLE_g_ and DLE_m_ are shown, respectively, on the 1^st^ and 2^nd^ lines. wMNE, sLORETA and dSPM are shown, respectively, on the 1^st^, 2^nd^ and 3^rd^ columns. The DLE_g_ maps are heterogeneous along the hippocampus shape. The spatial distribution has an increasing gradient from the hippocampus’s head to the tail. As shown, the DLE_g_ in the tail is more than double that in the head. DLE_g_ maps have similar patterns for the three methods, with the strongest values for sLORETA at the edges and the tail. DLE_g_ for wMNE give the best results, with errors below 0.8 cm in the majority of the head and the body. Conversely, DLE_m_ shows a strong mis-localization of the maximum for wMNE. The better DLE_m_ estimate is obtained for dSPM and sLORETA, where the error in the tail is the smallest. However, their spatial patterns are not similar. Indeed, dSPM has less DLE_m_ in the tail and edges and gives a homogeneous DLE_m_ map. In contrast, sLORETA, which also shows lower DLE_m_ in the head and the tail, shows a decreasing error in the body with respect to the source depth. DLE_s_ for amygdala are homogeneously distributed, with values close to the DLE_s_ values for the hippocampus’s head. The noise normalization impact of sLORETA and dSPM are largely the opposite. sLORETA has a lower DLE_m_ in the deeper central regions, such as the thalamus, with errors under 0.5 cm, whereas dSPM has a very good correction over the hippocampus and strong errors in the thalamus, where DLE_m_ ranges from 1 to 2 cm. Figure 5B shows histograms of DLE_g_ distributions for each method and for the five sizes of patches. These distributions are drawn in the case of the hippocampus. The distribution is similar for the three methods, with a better distribution of small errors (under 1 cm) for wMNE. Interestingly, activations of large patches give stronger DLE_g_, whereas large patches of 4 and 5 cm^2^ produce the strongest currents (see Table 1).

**Figure 6:**
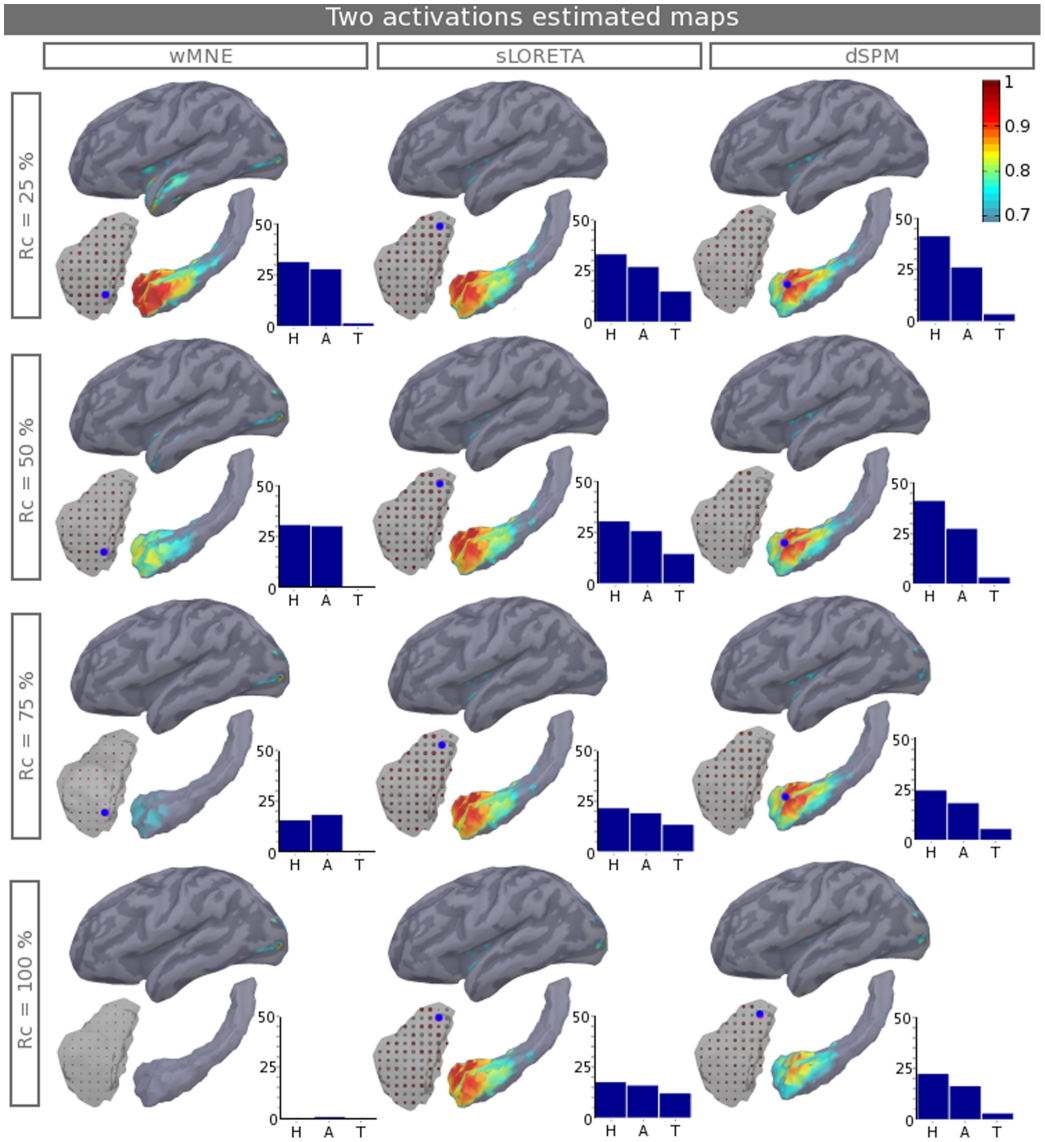
Two estimated maps of activations. Estimated normalized current maps for the three methods (columns). The actual patches are defined as shown in Figure 1, with one patch in the hippocampus and one patch in the neocortex. The results are given for the case of subject one; the values of D_t_ and the ratio (R_c_) are varied, where (R_c_) is the ratio between the neocortical activation and the hippocampal activation. A blue dot is displayed on the maps at the local estimated maximum in a sphere of 4 cm radius. The right bottom side of each map shows the histogram of the relative proportion (in percentage) of the estimated activations in the Hippocampus (H), the amygdala (A) and the thalamus (T). Note that the colorbar does not start from zero and that the structure sizes are modified to make the sources more visible. See Figure 1B for a medial view of the relative position of the structures.

Figure 6 shows the estimated maps for two simultaneous activations; one patch in the hippocampus and one neocortical patch (see their localization on Figure 1) for subject one by varying D_t_ and, thus, the ratio between the amplitude of the neocortical activation and the hippocampal activation. A dot colored in blue is displayed on the maps at the local estimated maximum in a sphere of 4 cm radius. This radius is, on average, half the distance between the sources of the hippocampus patch and the sources of the neocortical patch. The size of the activated patches is 3 cm^2^ for both the neocortex and the hippocampus. Four neocortical/subcortical ratios are shown; R_c_ equals 25, 50, 75 and 100%, respectively, from the 1^st^ to the 4^th^ line. Additionally, the right bottom side of each map displays the histogram of the relative proportion (in percentage) of the estimated activations in the hippocampus, the amygdala and the thalamus, to better quantify the well and badly localized activities by each method. When the hippocampal activity is stronger than the neocortex (R_c_ = 25%), hippocampal generators are well estimated by the three methods; there is a better spatial extent around the true patch for wMNE. However, wMNE shows some mislocalization in the lateral temporal cortex near the regions that have stronger cross-talk (see Figure 4), whereas the noise normalizations of both sLORETA and dSPM show less bias on the lateral temporal cortex but still significant values in the insula. Only dSPM maintains the maximum well localized in the hippocampus and has the highest proportion of activation in the hippocampus compared to the amygdala and thalamus. As expected, a higher increase in R_c_ causes the worsening of hippocampal localization for all of the methods. wMNE has good detection, which is up to R_c_ = 50% with no estimated sources in the thalamus. Conversely, for up to R_c_ = 100%, sLORETA and dSPM have good estimation; however, they create local maxima in the thalamus. This effect is stronger for sLORETA than for dSPM. The reconstructed activities in the visual neocortical patch are well defined for all three methods, but are not quantified here because they serve only as additional noise with variable amplitudes.

### 3.5 MEG Experiment

**Figure 7:**
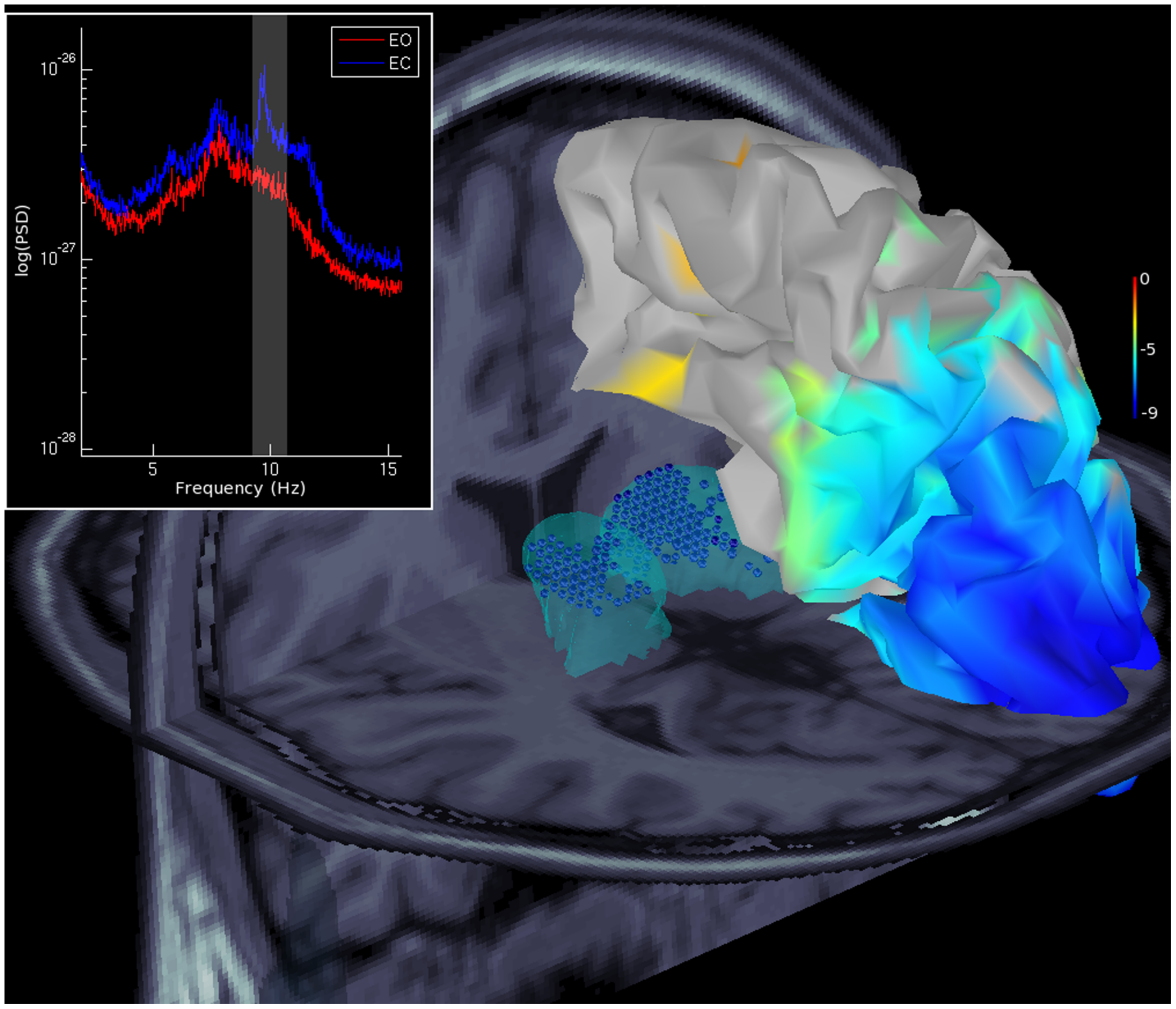
Thalamo-cortical modulations at rest. Thalamo-cortical modulations contrasting resting state eyes opened/closed conditions with 7 subjects using wMNE. The upper left rectangle displays the averaged power spectral density (PSD) of opened (red)/closed (blue) MEG data, with a vertical band (gray area) in the alpha frequencies. The right neocortex and the thalamic (light blue) envelopes are superimposed on the MNI T1 MRI. The overlaid neocortical map shows t values with a threshold at p<0.05, which are FDR corrected. Thalamic sources that pass the threshold are displayed with blue spheres, where the sizes are proportional to the estimated amplitudes.

The proposed experimental validation, based on resting state MEG data, allows us to detect thalamo-cortical modulations by contrasting eyes opened and eyes closed (EO/EC) conditions. The left upper white rectangle on Figure 7 displays the averaged spectral power densities (PSD) over all sensors and over the seven subjects. The power is the strongest during the eyes-closed condition (blue line) approximately 10 Hz. The figure also shows the EO/EC contrast from estimated sources in this alpha frequency band using wMNE. DBA detects strong bilateral source amplitude modulations in the posterior neocortical regions and in the thalami. For ease of reading, the right neocortex and the thalamic (light blue) envelopes are superimposed on the MNI T1 MRI. Thalamic sources passing the threshold are displayed as blue spheres, the sizes of which are proportional to the estimated amplitudes. The results obtained with the two other methods gave the same results.

## Discussion

This study aims to assess the source localization accuracy and to compare the characteristic of three inverse operators in the specific case of subcortical generators. This assessment was performed using the five steps that are discussed in detail in the following.

Using our realistic anatomical and electrophysiological model (DBA), we first show that one can expect magnetic fields that are detectable at the sensor level, especially for the hippocampus and amygdala. As shown on Figure 2A, this is explained by a compensation of the depth’s sources (whereas they have ten times less MEG sensitivity than the neocortex; see Figure 2B) by a realistic estimation of their neural currents, indeed, their current densities compensate the distance to the sensors even in the case of the amygdala, which have randomly oriented distributed dipoles. The thalamus has much lower simulated fields than other structures. Two reasons explain this result. The thalamus is considered in the DBA model to have a neural density that is ten times lower on average than the density in the neocortex and, thus, will produce smaller neural currents. Second, the thalamus has no preferential neural organization and is mainly composed of stellate cells; thus, we should expect more current cancellation within the structure. Consequently, thalamic activation could be detectable mainly by manipulating experimental paradigms to increase the signal to noise ratio (see the experimental validation in Figure 7) or by using an indirect measure, using hidden sources in Dynamical Causal Modeling [53]. Nevertheless, the increase in sensor numbers combining numerous gradiometers and magnetometers from the newest MEG systems accompanied by good hardware and software noise subtraction techniques could allow us to expect greater sensitivity to subcortical generators. Indeed, magnetometers have a higher sensitivity to deeper sources and, thus, could increase MEG sensitivity to deeper structures [54]. Concerning the special case of the hippocampus, the model used for now is a first approximation; we assume that pyramidal neurons composing the 3-layered archeo-cortex (Ammon’s horn, i.e., CA1 to CA4) [39] can be represented by the hippocampal external tessellation. Indeed, using 3T T1 MRI, we have not accessed the internal bilaminar structure and, thus, we cannot fully realistically estimate the cancellations between sub-territories or inside one sub-territory by increasing the size of the patch. Therefore, we decided to use the external envelope as a model and to consider that the main hippocampal generators are coming from the synchronous activation of Ammon’s horn macro-hippocampal column of pyramidal neurons. We hope, as a result of the high field 7 T MRI [55], [56], to improve this model and to evaluate the impact of the complex bilaminar geometry where the dentate gyrus is entangled with Ammon’s horn.

PSF and CTF results allow us to better understand how the three inverse operators manage the reconstruction of subcortical sources. PSF and CTF maps for hippocampus sources (see Figure 3 and Figure 4) give us a good overview of the regions from where (CTF) and to where (PSF) induce distortions, given the inverse operator. Considering a specific hippocampal point source using wMNE (see Figure 3), a regional map appears and moves in the temporal lobe according to its position. To extract a more global view of the most ambiguous regions at the whole hippocampal scale, Figure 4 shows regions that are obtained by averaging the PSF and CTF maps over all of the hippocampal point sources. These regions correspond to the nearest part of other subcortical structures and the nearest neocortical territories, the parahippocampal regions and the crests of the superior and inferior temporal sulci. In the two latter cases, the distortions could be the result of dipole orientations, which are very similar to the dipole orientations in most of the hippocampus. In contrast to wMNE, which has a high PSF on the lateral temporal lobe, the two other operators minimize the PSF distortion on the cortex. This correction is performed by construction and, in the case of sLORETA, the normalization appears to overcompensate for the correction in deeper sources with a strong biased spatial extent in the thalamus. Finally, cross-talk is found to be equal across the three methods. Indeed, the noise normalizations of sLORETA and dSPM modify only the point-spread functions. Cross-talk is maximal mainly for sources placed on the wall of the superior temporal sulcus. Accounting for this cross-talk is important in the interpretation of the estimated current maps because these regions will influence mostly the reconstruction of the hippocampal sources.

The error maps resulting from Monte Carlo simulations with the activations of single patches, i.e., D_t_ = 60 ms (see Figure 5A), are not homogeneously distributed along a given structure, especially for the hippocampus. These maps give us more spatial knowledge about the sub-territories that have the strongest errors, as is the case for the hippocampal tail and edges. Consequently, the results draw confidence maps for future subcortical source estimations. Globally, DLE_g_ shows better results using wMNE, with errors lower than 0.8 cm in the majority of the hippocampus and the amygdala. However, we can see on the hippocampus DLE_m_ maps that the noise normalization impact of dSPM and sLORETA is directly visible compared to wMNE. A significant improvement of both dSPM and sLORETA compared to wMNE is that because of their respective normalizations, they keep DLE_s_ ranges in the interval from 0.5 to 2 cm. Thus, the estimated maximum is still accurately localized with these two methods, whereas wMNE always mislocalized it. In contrast, sSPM and sLORETA show bias induced by the noise normalization. dSPM has an increase in DLE_m_ in the deepest regions, such as the thalamus, and sLORETA has an increase of DLE_m_ in the amygdala and the hippocampus body. Additionally, we can note that sLORETA should have zero localization errors [21] because this result was recently shown [22] in the case of a point source study using DLE_m_. However, this result is not the case in our DLE_m_ maps of single patch activations where Monte Carlo simulations are achieved using additive noise composed of real background activity and by using patches of activations that account for precise cortical/subcortical local morphology. We chose here to compute the head model using an overlapping spheres method, which is not optimal, especially in the case of deep sources. However, in a first approach, we wanted to assess our DBA model with the most popular method that is used routinely with our MEG data. In a second step, we will study in more detail the impact of the forward problem methods, using a more realistic model such as the BEM. Concerning the activations of single patches and by looking at the histograms of DLE_g_ (see Figure 5B), we can see a difference between small and large sizes of patches. Interestingly, activations of large patches give stronger DLE_g_. This result is paradoxical in the sense that the largest sizes of patches generate larger currents. However, because our model accounts for the geometry, the generated fields are more realistic. This relationship could be explained by the cancellation of currents that have opposite orientations. Similarly, Chupin et al. [57] show that magnetic fields generated by increasing sizes of patches are not linear and reach saturation with a patch size that is larger than 2 cm^2^. The closed shape and the small size of the hippocampus explain this phenomenon. This aspect is one more reason to better quantify, in the future, the resulting currents of hippocampus sub-territories using high field MRI.

Concerning two simultaneous activations, i.e., D_t_ <60 ms, the results show that the three methods could indeed localize hippocampal activation in the presence of a neocortical activation that has no more than 50% of the hippocampal amplitude. The simulated patch is in the hippocampal head, which is an area adjacent to the amygdala (see Figure 1B); this placement makes it very difficult to differentiate between these two parts. For this reason, the histograms show similar estimated currents in both the amygdala and the hippocampus. Moreover, wMNE has not completely compensated for the bias toward superficial sources and, thus, is more vulnerable to a region that has strong cross-talk. With a lower ratio, wMNE can no longer differentiate efficiently the subcortical generators from the neocortical generator. For a higher neocortical ratio, sLORETA and dSPM reconstruct hippocampal generators. However, these two methods produce local maxima in the thalamus, which could be mis-interpreted as another simultaneous activation. These false positives are the consequence of a higher spatial dispersion of the two noise-normalized methods compared to wMNE, as shown by the DLE_g_ results. When no strong assumptions are known, ambiguous configurations of neural generators appear with no possibility of determining which ones are real or not real. Consequently, depending on the experimenter’s questions, a trade-off should be made between using wMNE, which is more sensitive to cross-talk from superficial sources and does not reach a high level of detection, and using noise-normalized methods, which create deeper ghost sources. Moreover, similar to previous studies [6], [14], [15], by increasing the number of trials, improving the experimental paradigm or increasing the magnitude of neural currents, the localization accuracy will increase. The scope of our simulations is intrinsically limited by the chosen parameters, and several points should be discussed in more detail.

First, DMD that defines the activation amplitude plays a crucial role in the localization accuracy [58]. Choosing small DMDs most likely increases the DLEs. However, we decided to use the smallest DMDs given in the literature [13], and thus, our results show the worst case scenario. The hippocampal DMD might be increased to 0.8 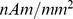, as shown in [59], instead of 0.4 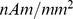,_ which was_ used in our simulations. In the same way, thalamic contributions are modeled with low DMD (ten times lower than the neocortex), which is an average approximation, and randomly oriented dipoles, which is a rough approximation of the cancellations occurring in this structure. Further studies are required to better estimate precisely the DMD and to define new ways to better approximate the neural thalamic architecture.

Second, the chosen SNR placed these simulations in the case of classical evoked field data. However, it could be interesting, as shown in the simulations of [15], to quantify the SNR threshold at which the method is no longer able to detect the simulated activities or how the DBA addresses ongoing oscillating data. Moreover, it could be interesting to define a lower SNR for subcortical sources than for the neocortex. Third, the impact of a higher patch size or a higher number of cortical sources [60] must be assessed in the subcortical case. Testing other imaging models could be a good starting point for further evaluations. Indeed, several studies developed inverse methods that combined parametric and distributed approaches based on multipole models [28], [61] and/or a multiresolution approach [62], [63] that better estimates spatial extension. These approaches could be adapted to volume-based structures such as the amygdala or thalamus (mainly composed of non-oriented cells). Finally, other parameters, such as the number of subjects or experimental paradigms, could also be evaluated.

In our experimental protocol, a large amount of data are recorded to contrast Eyes opened/Eyes closed conditions, in which cortico-thalamic modulations are strong enough to be detected. These results encourage us in our ability to detect subcortical activity, especially for regions that are assumed to contribute very weakly to MEG. However, the thalamic activations are found but are not accurate in the posterior parts of the thalamus, which is expected because of previous studies [47]. This result showed the limited spatial resolution that is sustainable by MEG in these very deep structures.
